# Breakdown-induced conductive channel for III-nitride light-emitting devices

**DOI:** 10.1038/s41598-018-34869-8

**Published:** 2018-11-08

**Authors:** Sang-Hyun Han, Seung-Hye Baek, Hyun-Jin Lee, Hyunsoo Kim, Sung-Nam Lee

**Affiliations:** 1 Department of Nano-Optical Engineering, Korea Polytechnic University, Siheung, 15073 Republic of Korea; 2 School of Semiconductor and Chemical Engineering, Semiconductor Physics Research Center, Chonbuk National University, Jeonju, 54896 Republic of Korea

## Abstract

III-nitride semiconductor-based light-emitting diodes (LEDs) have superior physical properties_,_ such as high thermal stability and brightness, for application to solid-state lighting sources. With the commercialization of GaN-based LEDs, improving LED reliability is important because they can be affected by electrostatic discharge, reverse leakage, and breakdown. However, research on the reverse bias characteristics of GaN-based LEDs is insufficient. We studied the reverse breakdown mechanism and demonstrated that a local breakdown can form a conductive channel in GaN-based LEDs, which can be expanded to a novel planar-type LED structure without an n-contact electrode. Furthermore, we found that this approach can be applied to AC-controllable light-emitting devices without any AC–DC converter.

## Introduction

Light-emitting devices (LEDv’s) have become one of the most widely used and most outstanding form of semiconductor diodes available today^1–4^. In particular, III-nitride-based semiconductors are promising materials for application in LEDv’s covering the ultraviolet and visible ranges of the electromagnetic spectrum^5–7^. Recently, significant advances have been made in nitride-based LEDs to realize a high optical output power for various solid-state lighting applications^8–10^. Many research groups have focused on optimizing the epi-structure, chip design, and packaging process to improve the optical and electrical performances^11–21^. Moreover, with regard to the n–p junction rectifier, the reverse breakdown and reverse leakage properties are major parameters for evaluating the LED performance and affect the electrostatic discharge damage and optical properties of LEDs^14,22,23^. The leakage current in GaN-based devices is substantial because GaN and its heterostructures are usually grown on foreign substrates such as sapphire, silicon, or silicon carbide, which results in a large number of defects and dislocations^11–13,24,25^. These crystal imperfections lead to not only a high reverse leakage but also an undesirable satellite luminescence, both of which are very different from the behaviour predicted by theory^26,27^. Therefore, there have been studies on understanding and improving the reverse breakdown and reverse leakage properties^28–36^. However, the reverse leakage current and breakdown mechanisms of GaN-based LEDs are still not clearly understood.

In this study, we found that the local reverse breakdown phenomenon plays a significant role in the formation of a carrier injection path in p–p and p–n–p GaN-based heterojunction structures. Moreover, we demonstrated the effectiveness of the p–n–p GaN-based LEDv when paired with a breakdown-induced conductive channel (BCC) obtained by using the local breakdown phenomenon, which can operate the p–n–p LEDv without an n-type electrode under DC and AC conditions. In particular, there are many requirements for operating LEDs under conventional AC conditions, such as the addition of an AC–DC converter, which would decrease the lifetime and increase the energy loss^20,21^. This novel approach to p-p and p–n–p LEDv’s has the potential to simplify the conventional fabrication process of n–p LEDs, including the lithography, etching, and n-metallization steps to form the n-type electrode for carrier injection^15,16^. We expect that the findings will provide an opportunity to advance novel planar-type p–n–p heterojunction optoelectronic devices without n-type electrode such as LEDs, photodetectors, and solar cells.

## Full breakdown and local breakdown in the III-nitride LEDs

**Figure 1:**
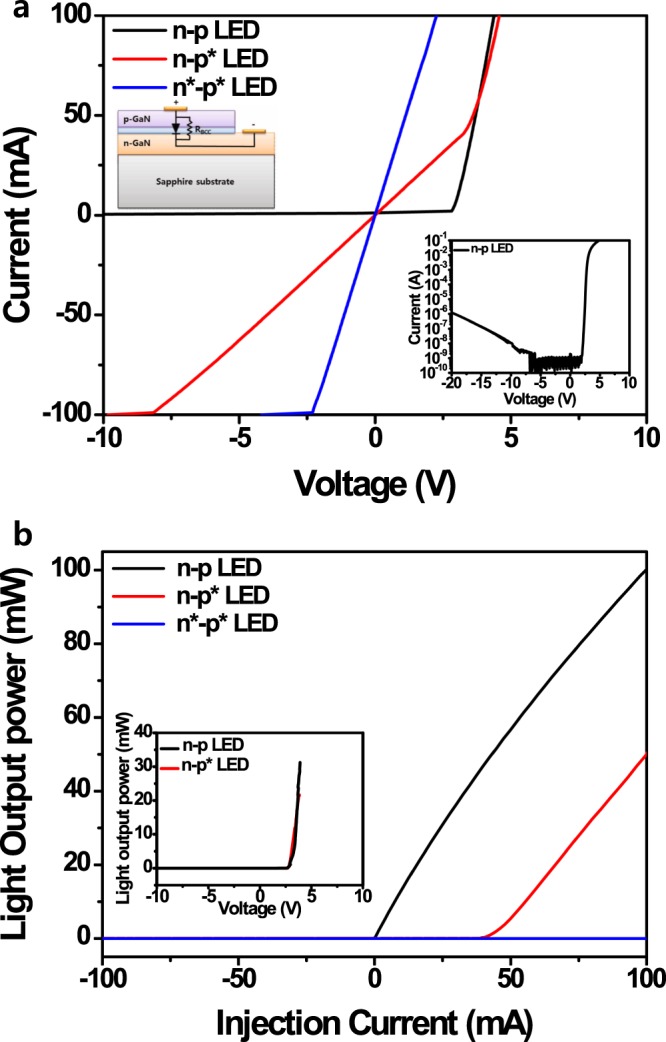
*L*–*I*–*V* characteristics of conventional n–p, n–p*, and n*–p* LEDs. (**a**) *I*–*V* and (**b**), *I*–*L* curves of conventional n–p, n–p*, and n*–p* LEDs. The insets of (**a**) show a schematic of the n-p* LED structure with BCC as a parallel resistance and the log I-V curve of n-p LED, and the inset of (**b)** shows the *V*–*L* curves of the n–p and n–p* LEDs.

Figure 1a,b show the current vs. voltage (*I*–*V*) and current vs. light output power (*I*–*L*) curves, respectively, of a conventional n–p LED under different breakdown conditions. The typical electrical behaviour of the n–p LED has been observed from *I*–*V* measurements to consist of a turn-on voltage (~2.54 V) and very low reverse leakage current (I_r_ = 0.45 nA at −5.0 V). After forming the breakdown at a reverse voltage (~100 V) much higher than the breakdown voltage of ~60 V, the breakdown LED indicates linear *I–V* characteristics and no emission properties. This is called the full breakdown n*–p* LED, where the asterisk represents the reverse breakdown. This is the very general breakdown phenomena in GaN-based LEDs^30,36,37^. However, after a local breakdown (p*) is formed in the only p-layer under the reverse breakdown voltage of ~60 V, the *I*–*V* curve of the n–p* LED represents almost linear properties—like a conductive material—below +3.4 V and then the normal *I*–*V* characteristics of an n–p LED above 3.4 V. As a result, the electrical properties of the n–p* LED were modified from an n–p diode to an n–n- or n–i-like structure in the low-voltage region. This led to almost linear *I*–*V* properties like a full breakdown n*–p* LED below a turn-on voltage of 3.4 V. Based on these results, we believe that the limited linear *I–V* characteristics of the n–p* LED can be attributed to the formation of a BCC in the local breakdown p*-layer, as shown in Fig. S1b. However, as the forward voltage was increased above 3.4 V, the *I–V* properties of the n–p* LED represented the same *I*–*V* characteristics as an n–p LED. We surmised that the injection current through the BCC became saturated within the limited BCC around 3.4 V (Fig. S1c) and overflowed to other non-breakdown regions above 3.4 V (Fig. S1d). This means that the *I–V* characteristics of the n–p* LED recovers from the breakdown n*–p* to an n–p LED above a critical voltage (>3.4 V) because the BCC can bypass the current in the n–p* LED like a parallel resistance (R_p_), as shown by the inset of Fig. 1a.

The electroluminescence (EL) of an n–p* LED would not be observed at a low injection current (<40 mA), which was the saturation point of the carrier flow through the BCC and the starting position of the carrier overflow from the BCC to non-breakdown regions at ~3.4 V. This led to radiative recombination from the active layer, as shown in Fig. S1d. During carrier injection through the BCC (<3.4 V), radiative electron–hole recombination did not happen at the region of InGaN/GaN quantum wells (QWs), which is consistent with the large forward leakage current below 3.4 V in the n–p* LED. This indicates that the BCC acting as an R_p_ in the current bypass was formed at the p-type layer and InGaN active region. However, the EL intensity of the n–p* LED exhibited almost the same emission intensity behaviour as that of the n–p LED at different applied forward voltages, as shown by the inset of Fig. 1b. This indicates that the applied voltage can control the carrier flow and emission performance of nitride-based n–p* LEDs. Based on these results, we suggest that the reverse breakdown phenomenon forms a localized BCC in nitride-based LEDs, which can play a role in a limited current-leakage path under a low forward bias (<3.4 V).

## Formation of BCC and giant dot-like luminescence from surface V-defect

**Figure 2:**
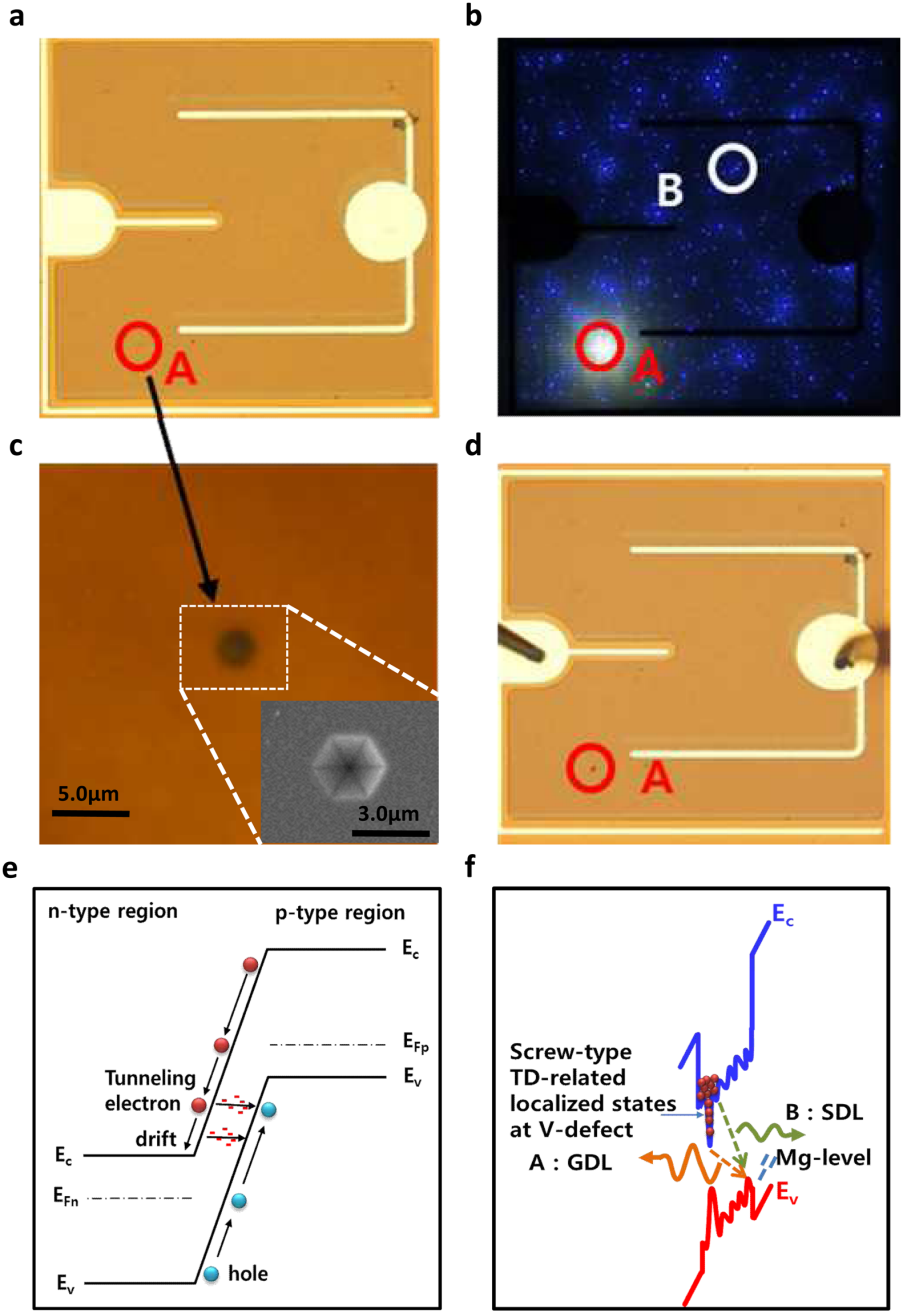
Optical microscope images of GaN-based LEDs with different reverse voltages and schematic diagrams of the reverse tunnelling-assisted transport leakage path. (**a**) Optical microscope image of the as-fabricated n–p LED before formation of the BCC. (**b**) Reverse bias-induced dot-like luminescence image at the reverse bias of −25 V showing two kinds of dot-like luminescence: SDL at position B and GDL at position A. (**c**) Magnified optical microscope image of the surface V-defect. The inset of c is the SEM image of the surface V-defect that can generate GDL. (**d**) Optical microscope image of the n–p* LED after formation of the BCC. (**e**) The band diagram of tunnelling-assisted reverse leakage process. (**f**) The emitting transport paths of the conventional SDL and GDL in the InGaN active region.

Figure 2a,b show the as-fabricated LED image and its reverse dot-like luminescence under the reverse voltage of 25 V, respectively. There are two kinds of dot-like luminescence: the general small dot-like luminescence (SDL) at position B and the giant dot-like luminescence (GDL) at position A. As the reverse breakdown voltage was increased to ~60 V, we observed the local breakdown phenomena to be a BCC at position A, as shown in Fig. 2d. Based on these results, we believed that the GDL region induced by the reverse bias can form a local breakdown to be form a BCC in an n–p LED. In addition, we initially could not clearly observe any surface defect at position A using an optical microscope because of its very small size (<3.0 µm), as shown in Fig. 2a. However, after we measured the GDL region, we accidentally found a surface V-defect at position A, as shown in Fig. 2c. It implied that the GDL is generated by the surface V-defect region as the reverse bias is increased, as shown in Fig. S2. Therefore, we suggest that the surface V-defect of a GaN-based LED can generate a GDL at a reverse bias and that the BCC can be formed at surface V-defect as the reverse bias reaches the breakdown voltage.

Figure 2e,f show schematic diagrams of the reverse tunnelling-assisted transport leakage path and the emitting transport paths of the conventional SDL and GDL in the InGaN QW region, respectively. We observed that the GDL among the reverse dot-like luminescences originated from the surface V-defect, which is the main source of the reverse breakdown phenomenon for forming a BCC. In general, defect-related leakage carriers are injected to the QW through preferential paths with a lower potential barrier, which leads to the SDL^38^. The surface V-defect that formed at the threading dislocation represents a small valley edge region with a higher In content than the sidewall regions like a quantum dot^39^; this leads to the GDL transiting from the deeper localized state of the V-defect to the lowest energy level of the valence band. Because the deeper localized state formed in the valley end of the surface V-defect can easily be filled by the injection carrier, we suggest that this deep level transition first emits at the valley end, and the emission of the V-defect expands from the valley end to the sidewalls with six facets of {10–11}. This results in the GDL, as shown in Fig. S2.

## Relationship between reverse leakage current and BCC

**Figure 3:**
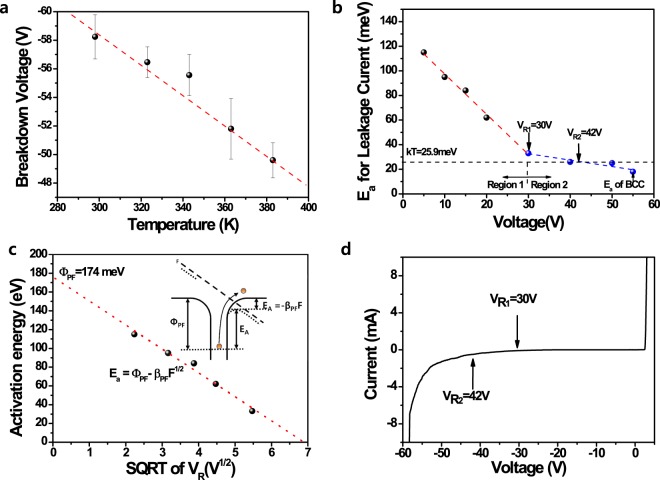
Temperature dependence of the breakdown voltage and the thermal activation energy of the reverse leakage current in GaN-based LEDs with the different reverse voltages. (**a**) Reverse breakdown voltages of GaN-based LEDs as a function of temperature. (**b**) Activation energy of the reverse leakage current in GaN-based LEDs as the reverse voltage is increased from 5 to 50 V. (**c**) The activation energy of the leakage current at a low reverse bias (<30 V) as a function of the square root of the reverse voltage. (**d**) The room temperature I-V curve of GaN-based LED.

In order to understand the local breakdown mechanism to form a BCC, we measured the temperature-dependent breakdown voltage of the GaN-based LED. In general, the breakdown voltage V_B_ at a temperature T is given by the relation

1 $${{\rm{V}}}_{B}={{\rm{V}}}_{{\rm{B}}0}[1+{\rm{\beta }}({\rm{T}}-{{\rm{T}}}_{0})],$$

where V_B0_ is the breakdown voltage at room temperature, T_0_ is the room temperature, and β is the temperature coefficient of the breakdown voltage^31,32^. Figure 3a shows that the temperature coefficient (β) of the breakdown voltage had a negative value as the measured temperature increased. It indicates that the BCC formation mechanism is a Zener-type breakdown phenomenon^31,32^. In particular, because the BCC can be generated at a surface V-defect, we suggest that the BCC is formed by the defect-assisted Zener breakdown mechanism. We also obtained the thermal activation energy (E_a_ = 18.6 meV) to form a BCC at the temperature-dependent breakdown voltage of GaN-based LEDs (Fig. 3a), which is lower than the thermal energy (kT = 25.9 meV) at room temperature. This implies that the reverse-bias-induced Zener breakdown can occur more easily than the escape of a thermally assisted carrier. Figure 3b shows the activation energies of the reverse leakage current and breakdown voltage in the GaN-based LED as a function of the reverse voltage. Two slopes of activation energies were found for the reverse voltage, which indicates two reverse leakage mechanisms: (1) a low reverse bias region below 30 V and (2) high reverse bias region above 30 V. The activation energy of the temperature-dependent reverse leakage current for the carriers trapped in deep centres to escape can be represented by an Arrhenius plot (Eq. 2), which can be expressed as follows^29,40^:

2 $${\rm{I}}\propto \exp (-{{\rm{E}}}_{{\rm{a}}}/{\rm{kT}})$$

3 $${{\rm{E}}}_{{\rm{a}}}={{\rm{\Phi }}}_{{\rm{PF}}}-{{\rm{\beta }}}_{{\rm{PF}}}{{\rm{F}}}^{1/2}$$

where E_a_ is the thermal activation energy, k is the Boltzmann constant, Φ_PF_ is the barrier height of the carrier trapped in the deep centre without an external electric field, β_PF_ is the Poole–Frenkel constant, and F is the local electrical strength applied to the deep centres shown in inset of Fig. 3c. This indicates that the activation energy of the reverse leakage current decreases with an increasing reverse bias because of the increase in the reverse bias-induced electric field strength (F) at deep levels. Figure 3c shows the activation energy of the leakage current at a low reverse bias (<30 V) as a function of the square root of the reverse voltage. This indicates that the leakage current of region 1 can be dominated by the Poole–Frenkel emission, which has a Poole–Frenkel barrier (Φ_PF_) of 174 meV and constant slope of β_PF_. The electric field (F) may be increased by different deep centres that can be formed by many crystal defects or the In composition fluctuations of the InGaN active layer; this indicates the possibility of extending the Poole–Frenkel region up to ~30 V within region 1. The activation energy (E_a_ = 18.6 meV) for the BCC formation breakdown voltage was an extension of the second activation energy fitting curve in region 2. It indicates that the BCC-related reverse breakdown is closely related to another reverse leakage mechanism. In particular, the reverse leakage current is reported to be strongly related to the space-charge limited current above the region of the Poole–Frenkel emission^40,41^. Moreover, the V_R2_ (~42 V) at which the fitting curve contacted the thermal energy (kT = 25.9 meV) is almost consistent with the soft breakdown. Based on these results, we suggest that a BCC can be formed by the defect-assisted Zener breakdown, which can be generated from a soft breakdown related to the space-charge limited current after the Poole–Frenkel leakage model is closely followed at the low-voltage region 1.

## p-n-p*LEDv without n-type electrode

**Figure 4:**
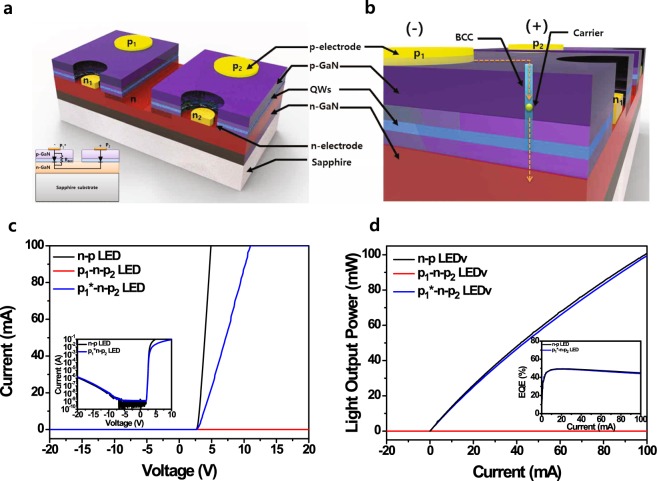
Schematic structures and *L*–*I*–*V* characteristics of the GaN-based n–p LED and p–n–p* LEDv without an n-contact electrode. **(a**) Schematic diagram of the fabricated nitride-based p–n–p LEDv consisting of two conventional n–p LEDs separated by an n-type mesa area. The inset of (**a**) shows schematic diagrams of the cross-section structure of the p_1_*–n–p_2_ LEDv without the n-contact electrode. (**b**) Schematic diagram of the carrier transport through the BCC in the p* region of the p–n–p* LEDv. (**c**) *I*–*V* and (**d**) *I*–*L* curves of the n–p LED and p_1_–n–p_2_ and p_1_*–n–p_2_ LEDv’s. Insets of (**c**,**d)** show the log I-V curves and the external quantum efficiency (EQE) of the n–p LED and p_1_*–n–p_2_ LEDv’s, respectively.

Figure 4a depicts the schematic of a p_1_–n–p_2_ LEDv consisting of two n–p LEDs with n-type (n_1_ and n_2_) and p-type electrodes (p_1_ and p_2_). This p_1_–n–p_2_ LEDv was systematically evaluated by comparing with the n_1_–p_1_ and n_2_–p_2_ LEDs because they share the same epi-structure and chip design. In an n-p LED, the n-type (n_1_) and p-type (p_1_) electrodes are injected with cathode and anode currents, respectively. However, the p_1_*- and p_2_-electrodes of the p_1_*–n–p_2_ LEDv are introduced to the cathode and anode currents, respectively, without the use of an n-type electrode as the cathode, as shown in Fig. 4b. In terms of the carrier path, therefore, the conventional LED is an n_1_–p_1_ or n_2_–p_2_ structure, whereas the BCC-embedded LEDv is a p_1_–n–p_2_ structure whose p-electrodes are separated by the etched n-type region to form the isolation between two n_1_–p_1_ and n_2_–p_2_ LEDs.

Figure 4c shows the *I*–*V* characteristics of an n–p LED, the p_1_–n–p_2_ and p_1_*–n–p_2_ LEDv structures. The reverse breakdown of the n–p LED with a turn-on voltage of 2.94 V did not occur until −20 V, which indicates the excellent crystal properties. On the other hand, when the anode and cathode currents were applied to the p_1_- and p_2_-electrodes, respectively, in the p_1_–n–p_2_ LEDv without a BCC, there were no forward and reverse currents as we expected. However, the *I*–*V* curve of the p_1_*–n–p_2_ LEDv with the BCC in the p_1_ region showed characteristics similar to the *I*–*V* curve of the n–p LED because the injection current could be transferred to the p_2_ region through the BCC in the p_1_* region. In addition, the reverse leakage currents of n–p LED and p_1_*–n–p_2_ LEDv are 0.45 nA and 1.34 nA at the reverse bias of − 5.0 V, respectively. It indicates that the leakage current of p_1_*–n–p_2_ is not significantly affected by BCC formation. However, above the turn-on voltage, the series resistance of the p_1_*–n–p_2_ LEDv was higher than that of the n–p LED owing to the increase in carrier paths through the p*-region and n-type region between the p_1_* and p_2_ electrodes. Figure 4d shows the light output power of the n–p LED, the p_1_–n–p_2_ and p_1_*–n–p_2_ LEDv’s as a function of the injection current. The EL emission of the n–p LED exhibited a significant increase above the turn-on bias of 2.94 V, whereas that of the p_1_–n–p_2_ LEDv was not observed within our measurement range. However, the p_1_*–n–p_2_ LEDv showed *I*–*L* behavior similar to that of the n–p LED. From these results, we achieved that the external quantum efficiencies of conventional n-p and p_1_*–n–p_2_ LEDs were 49.5% and 48.8% at the injection current of 20 mA, respectively, as shown in inset of Fig. 4d. It indicates that the p_1_*-layer plays a role in the supplemental path of electrons as a parallel resistance under cathode-injection conditions, as shown in the inset of Fig. 4a. For these reasons, we believe that the injected electrons move from the p_1_*-layer to the p_2_-layer through the n-layer in the p_1_*–n–p_2_ LEDv. This leads to similar *L*–*I–V* characteristics to an n-p LED like a low turn-on voltage and strong EL emissions from the p_2_ region of the p_1_*–n–p_2_ LEDv.

## AC-controllable p*-n-p*LEDv

**Figure 5:**
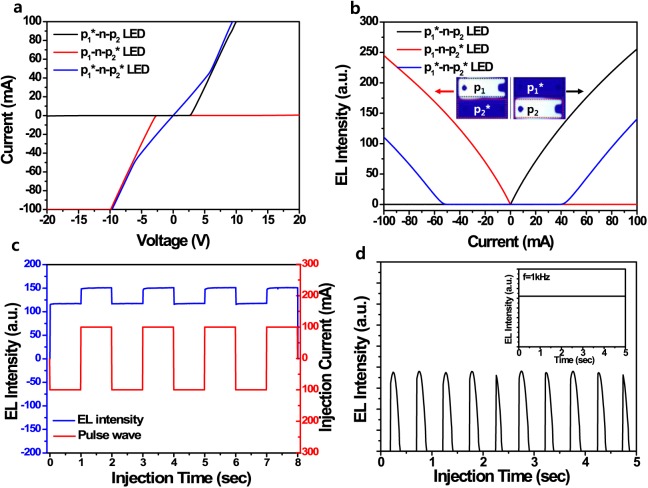
*L*–*I*–*V* characteristics of the BCC-embedded p_1_*–n–p_2_* LEDv under positive and negative bias conditions and AC frequency conditions. (**a**) *I*–*V* and (**b**), *I*–*L* curves of the BCC-embedded p_1_*–n–p_2_, p_1_–n–p_2_*, and p_1_*–n–p_2_* LEDv’s. The inset of b shows the EL images obtained from the p_1_–n–p_2_* and p_1_*–n–p_2_ LEDv’s at an operating current of ±100 mA. (**c**) EL emission intensity of the p_1_*–n–p_2_* LEDv as a function of the pulse injection time (s) (**d**), EL intensity of the p_1_*–n–p_2_* LEDv as a function of the AC injection time at a constant peak V_p_ of 7.0 V under different AC frequencies of 1 Hz and 1000 Hz.

Figure 5a shows the *I*–*V* curves of the p_1_*–n–p_2_, p_1_–n–p_2_*, and p_1_*–n–p_2_* LEDv’s, which were obtained by using only p-type electrodes as the anode and cathode currents without the n-type electrode. In addition, we measured the *L*–*I* curves of the p_1_*–n–p_2_, p_1_–n–p_2_*, and p_1_*–n–p_2_* LEDv’s by changing the current direction from the p*-layers, as shown in Fig. 5b. Before forming the BCCs in the p-layers, we did not observe the turn-on voltage and emission at applied voltages of –20 to 20 V because of a huge Schottky barrier for negative bias and the formation of depletion regions between the p_1_–n and n–p_2_ junctions in the p_1_–n–p_2_ LEDv. However, after BCCs were formed in the p_1_–n–p_2_ LEDv by using the current-induced breakdown methods shown in Fig. S3a, we found that the p_1_*–n–p_2_ and p_1_–n–p_2_* LEDv’s exhibited *I*–*V* curves similar to that of a typical p–n LED consisting of a turn-on voltage and series resistance. In the p_1_–n–p_2_* LEDv, when we applied the anode and cathode currents to the p_1_ and p_2_* electrodes (Fig. S3c), respectively, the turn-on voltage was ~2.75 V, and the reverse breakdown was not observed up to –20 V. Similarly, when we applied the cathode and anode currents to the p_1_* and p_2_ electrodes in the p_1_*–n–p_2_ LEDv, the turn-on voltage was ~2.67 V, and the reverse breakdown was not observed up to +20 V. We clearly observed the EL emission from p-layer of the p*–n–p LEDv’s by applying the anode and cathode currents to p- and p*-layers, respectively. Because the p_1_*–n–p_2_ and p_1_–n–p_2_* LEDv’s operated as n–n–p_2_ and p_1_–n–n LEDv’s, respectively, both EL emissions were only observed from the p_2_ and p_1_ regions, respectively, as shown in the inset of Fig. 5b.

However, when the BCCs were formed in both p-layers (Fig. S3d,e), there was no turn-on voltage for the p_1_*–n–p_2_* LEDv. In the applied voltage range of –6.0 to +5.9 V, the *I*–*V* curve showed linear resistance properties similar to those of the resistance of a conductive material. However, as the positive bias (>|6.0| V) to the p_1_* and p_2_* electrodes increased, the p_1_* and p_2_* layers recovered to the original properties of p_1_ and p_2_ layers as the source of positive carriers because of the saturation of the current flow through the limited BCCs. In particular, the p_1_*–n–p_2_* LEDv exhibited both side emissions of the p_1_* and p_2_* regions at the injection currents of >42 mA and <–50 mA, respectively. However, there was no EL emission between –50 and 42 mA, which is consistent with the linear region (−6.0 to +5.9 V) of the *I–V* curve. These results indicate that the EL emission of the p_1_*–n–p_2_* LEDv is alternatively generated from both the p_1_* and p_2_* layers by the directions of the injection bias. As a result, we observed the EL emissions of the p_1_*–n–p_2_* LEDv under the pulse conditions (pulse width of 1 s and 50% duty cycle) of ±100 mA operating current. As the p_1_*–n–p_2_* LEDv was injected with the pulsed current, the EL emission was observed similar to the pulse injection condition, as shown in Fig. 5c. This indicates that the p_1_*–n–p_2_* LEDv can be consistently operated by an alternating bias for continuous EL emissions under the pulsed injection conditions. However, the positive-bias-induced EL intensity of the p_1_*–n–p_2_* LEDv was slightly higher than the reverse-bias-induced EL intensity shown in blue line of Fig. 5c. This may be due to the difference in the BCCs of p_1_* and p_2_*, such as the size and position. This is consistent with the slightly asymmetric breakdown-induced voltage-drop phenomenon and *I*–*L* characteristics (blue line) shown in Figs S3a and 5b, respectively. In addition, we observed the AC performance of the p_1_*–n–p_2_* LEDv as the AC frequency was increased. Figure 5d show the EL intensity of the p_1_*–n–p_2_* LEDv under different AC conditions (the peak voltage, V_p_ = 7.0 V) from 1 Hz to 1 kHz as a function of time. The EL intensity of the p_1_*–n–p_2_* LEDv matched the operating voltage of the AC frequencies well. Despite the AC frequency being increased to 1000 Hz, the EL emission of the p_1_*–n–p_2_* LEDv operated well, as shown in inset of Fig. 5d. Furthermore, we clearly observed that the light was alternately emitted from p_1_* and p_2_* regions at 1.0 and 1000 Hz AC frequencies shown in Fig. S4. This indicates that the p_1_*–n–p_2_* LEDv without an n-electrode can be used for AC lighting sources without an AC–DC converter.

## Conclusions

We studied the local breakdown phenomenon and its applications for an n–p and p–n–p GaN-based LEDv’s by applying the critical bias to the p-type layers. When a reverse bias was applied to the n–p LED, it found that BCCs were formed in the surface V-defect to transport carriers in the breakdown region (p*) of the n–p LED. The temperature-dependent breakdown voltages of the n–p LEDs showed that the BCC formation mechanism was the defect-assisted Zener breakdown phenomenon. In addition, the p_1_*–n–p_2_* LEDv exhibited both side emissions of the p_1_* and p_2_* regions at the alternative injection currents. Based on the results, we suggest that this approach promises a novel n-type electrode free p–n–p* LEDv and an AC-controllable p*–n–p* LEDv without an AC-DC converter, leading to the extension to additional new applications.

## Methods

We prepared a 2.0-µm-thick (0001) GaN template grown on a *c*-plane sapphire substrate by using a conventional two-step growth method employing metal–organic chemical vapor deposition (MOCVD) developed in-house^2^. Trimethylgallium (TMGa), trimethylindium (TMIn), and ammonia (NH_3_) were employed as the Ga, In, and N sources, respectively. Silane (SiH_4_) and biscyclopentadienylmagnesium (Cp_2_Mg) were used as the n- and p-type dopants, respectively. After growing the *c*-plane GaN template, we grew a conventional n–p heterojunction LED structure consisting of a 3-µm-thick Si-doped n-type GaN layer (n_e_ = 2.0 × 10^18^/cm^3^), five-period InGaN/GaN quantum wells (QWs), and a 0.1-µm-thick Mg-doped p-type GaN layer (n_h_ = 1.0 × 10^18^/cm^3^). After the LED wafers were grown, two types of LED chips were produced in a standard fabrication process: a conventional n–p LED with lateral electrodes and a novel p–n–p LEDv with two p-electrodes separated by a mesa structure consisting two conventional lateral-electrode-type LEDs (see Fig. 4a). In the structures of the conventional n–p LED and novel p–n–p LEDv, the n-type and p-type metals were Ti/Al and Ni/Au, respectively, and deposited by an electron-beam evaporator. Before depositing the p-type metal, we deposited a 100-nm-thick indium–tin oxide layer on p-GaN as a transparent conductive electrode to increase the current-spreading effect. To measure the local breakdown phenomenon in the p-layer of the n–p LED and p–n–p LEDv, we evaluated the voltage and current of both the LED and LEDv by using a Keithley 2400 source meter. The *L*–*I*–*V* measurements were performed with an HP-4155 parameter analyser (Hewlett-Packard, now Agilent Technologies). In particular, we observed SDL and GDL of the n-p LED using a high-magnification objective lens under reverse bias. The surface V-defect of GaN-based LED was analysed by a scanning electron microscopy.

## Electronic supplementary material

Supplementary information

Figure S4(a)-video

Figure S4(b)-video

Figure S4(c)-video

Figure S4(d)-video
