# Supplementary material for: Breakdown-induced conductive channel for III-nitride light-emitting devices
Source: Sci Rep. 2018 Nov 8;8:16547. doi: 10.1038/s41598-018-34869-8 (PMC6224508; doi:10.1038/s41598-018-34869-8)
Supplement: Supplementary file 1 — Supplementary information [file 41598_2018_34869_MOESM1_ESM.docx]

**Supplementary Information**

**Breakdown-induced conductive channel for III-nitride light-emitting devices**

Sang-Hyun Han^1^, Seung-Hye Baek^1^, Hyun-Jin Lee^1^, Hyunsoo Kim^2^ & Sung-Nam Lee^1^*

^1^*Department of Nano-Optical Engineering, Korea Polytechnic University, Siheung 15073, Republic of Korea*

*^2^School of Semiconductor and Chemical Engineering, Semiconductor Physics Research Center, Chonbuk National University, Jeonju 54896, Republic of Korea*

**S1. The carrier-transport and luminescence process through BCC in the n–p* GaN-based LED**

**S2. Analysis of GDL generated at the surface V-defect in the GaN-based LEDs with increasing reverse bias**

**S3. Formation method of BCC in the GaN-based p*-n-p* light emitting devices**

**S4. AC-frequency dependent EL analysis of the BCC-embedded p_1_*–n–p_2_* LEDv**

**S1. The carrier-transport and luminescence process through BCC in the n–p* GaN-based LED**


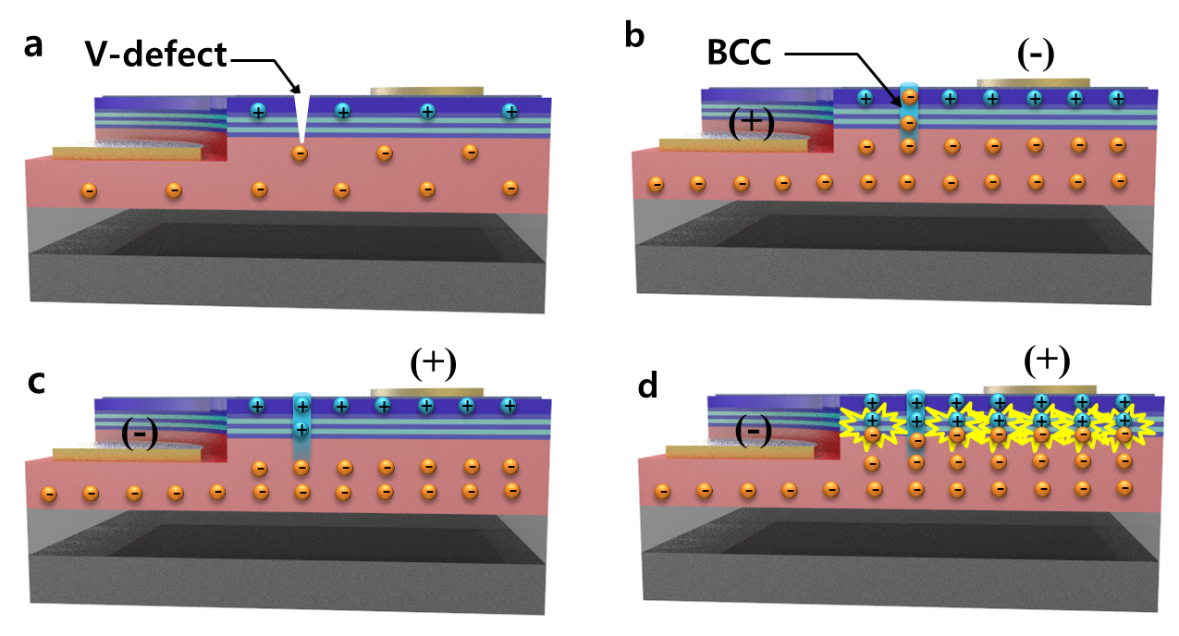


**Figure S1** The carrier-transport process of the n–p* LED is modelled in (a-d). (**a**) GaN-based n-p LED, (**b**) the formation of BCC under the reverse breakdown, (**c**) the large leakage current through BCC at the forward bias, (**d**) the forward emission from the non-breakdown region after saturation of forward current through BCC.

In the GaN-based LED, the BCC can be generated at the surface V-defect region under the reverse breakdown condition as shown in Fig. S1a and b. The BCC of p-layer represents the localized conductive behaviour in the p*-layer, indicating that the injection current can be bypassed the active layer as a parallel resistance. However, as forward bias is increased, the transport of majority carriers through limited BCC in the p*-layer becomes saturated and the original p-type original properties were recovered, as shown in Fig. 1Sd, leading to the *I*–*V* characteristics and EL emission similar to the those of a conventional n–p LED above the turn-on bias shown in Fig. 1a.

**S2. Analysis of GDL generated at the surface V-defect in the GaN-based LEDs with increasing reverse bias**

**
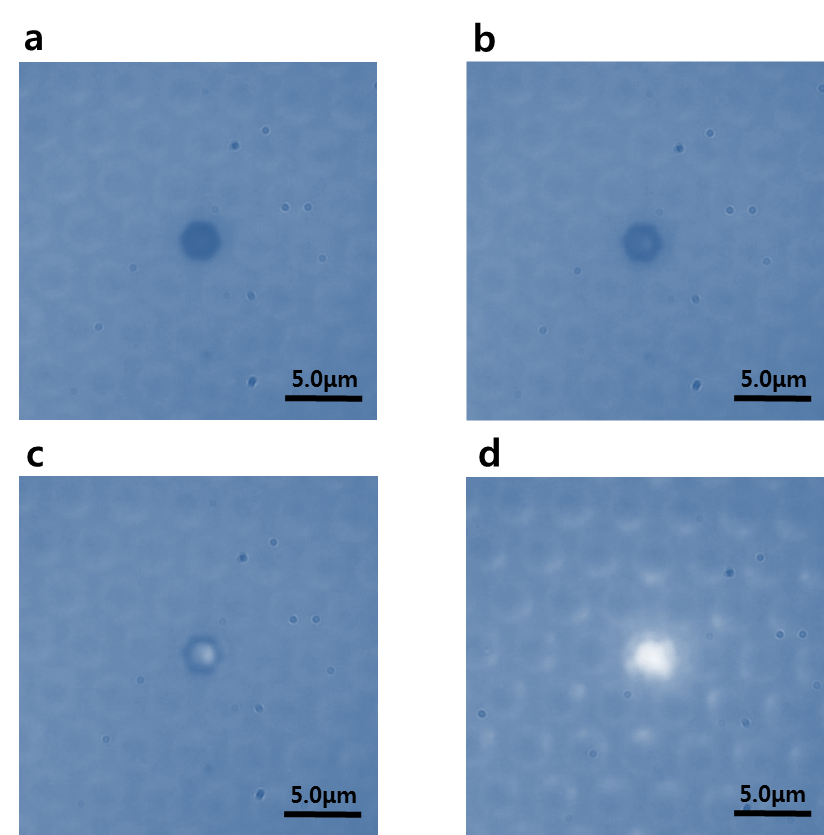
**

**Figure S 2** Micro-EL images of GDL in the surface V-defect of the GaN-based LEDs with increasing reverse bias. The GDL is clearly observed from the surface V-defect region as the reverse bias (**a**) -15V, (**b**) -18V, (**c**) -21V and (**d**) -24V increase.

We clearly observe the surface V-defect on GaN-based LED grown on patterned sapphire substrate shown in Fig. S2a. One can see that, when applying reverse bias, the emission images of GDL was found in the surface V-defect of the GaN-based LED using the high-resolution optical microscopy. After the GDL occurs at the valley-end of the surface V-defect at -15 V, the GDL extends from valley-end to the sidewall as the reverse voltage increases shown in Fig. S2 b-d. From these results, we demonstrate that the GDL can be generated from the surface V-defect, which can form BCC in the GaN-based LEDs.

**S3. Formation method of BCC in GaN-based p*-n-p* light emitting devices**


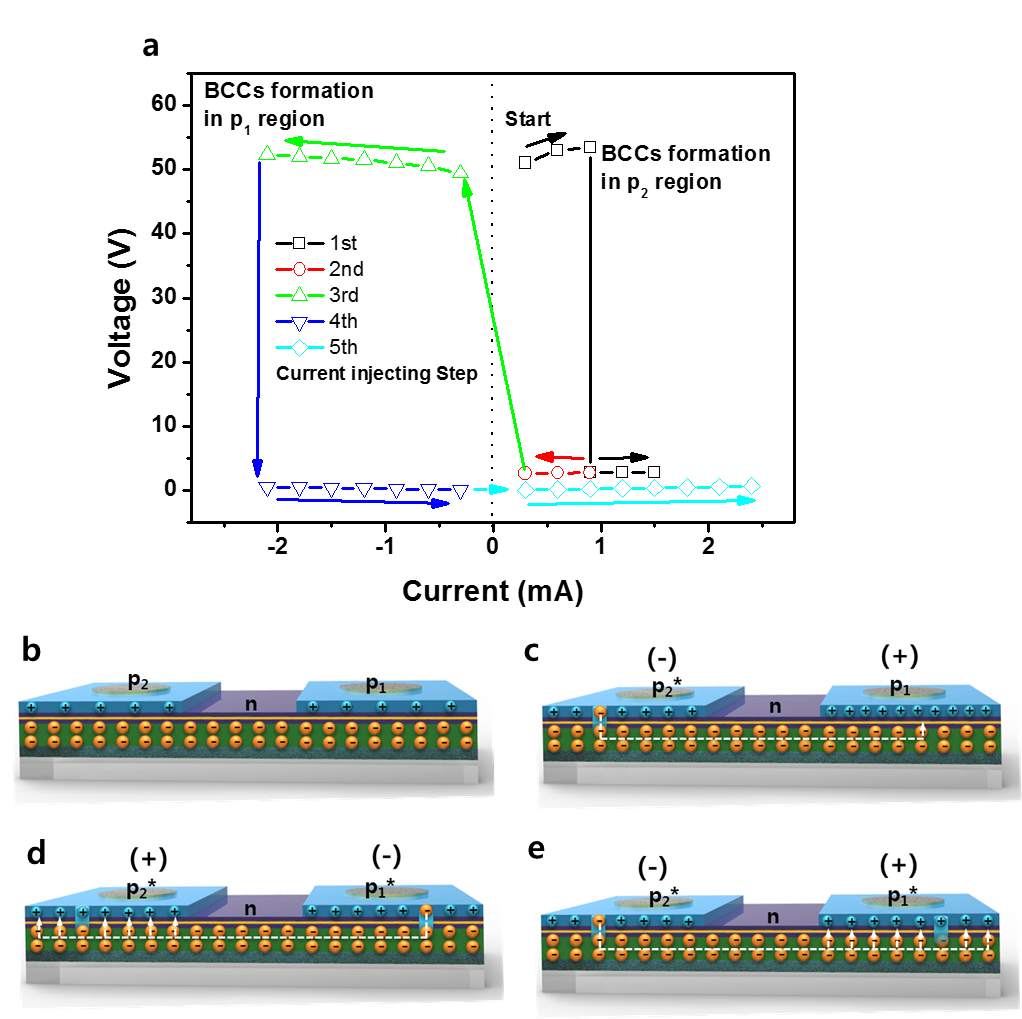


**Figure S 3** *I*–*V* characteristics and schematic diagram of BCC formation of the p_1_*–n–p_2_* LEDv. (**a**) The *I*–*V* characteristics of the p_1_–n–p_2_ LED structure after alternatively introducing an anode and a cathode currents to two p-type layers. (**b**) the p_1_–n–p_2_ LEDv structure without applied bias conditions, (**c**) the first injection condition (1^st^ and 2^nd^ steps in a) shows that the anode and the cathode current were applied to the p_1_ and p_2_ layers, respectively, whereas the reverse direction condition (3^rd^ and 4^th^ steps in a), (**d**) indicates that the anode and the cathode currents were applied to the p_2_ and p_1_ layers, respectively. (**e**) in 5^th^ step, this voltage-drop phenomenon may have been maintained by forming BCC in the p_1_*–n–p_2_* LEDv.

In order to clarify the electrical behaviour of BCC-formation in the p–n–p LEDv structure, we measured the *I*–*V* characteristics of the p_1_–n–p_2_ LED structure (Fig. S3b) after introducing alternatively an anode and a cathode current to two p-type layers, one after the other in the p_1_–n–p_2_ heterojunction. We first applied the anode and the cathode currents to the p_1_ and p_2_ electrodes, respectively (Fig. S3c). As the low injection current (~0.3 mA) was applied, a very high operation voltage of up to –50 V was observed, which was similar to the behaviour of the conventional n–p LED under reverse bias. However, as the applied current was increased above 0.9 mA, the operation voltage of the p_1_–n–p_2_ LED structure suddenly decreased from 53.5 to 2.75 V. This voltage-drop phenomenon continued as the applied current was increased and remained even after the injection current was reduced below 0.9 mA, as shown in the 2^nd^ step in Fig. S3a, indicating that the BCC was formed in the p_2_ region as a current path of electrons. As a result, we believe that the electrical properties of the p_1_–n–p_2_* structure would be modified to resemble those of the p_1_–n–n- or p_1_–n–i-like structure under this injection condition owing to the supply of electrons through the BCC in the p_2_ region. From these results, we consider that the carrier transport in p_1_–n–p_2_* was easy enough to lower the operation voltage in the 2^nd^ step. On the contrary, we applied the anode and the cathode currents to the p_2_* and p_1_ electrodes, respectively (Fig. S3d). In the region near the applied current (–2.0 mA), the operation voltage of the p_1_–n–p_2_* structure was again increased to –53 V owing to the reverse bias to the p_1_ electrode for the cathode current, which then gave rise to the voltage-drop phenomenon at an applied current of below –2.0 mA owing to the BCC formation in the p_1_ region as the injection current was increased. In this step, the BCC-embedded p_1_* region supplied negative charges from the cathode current to the p_2_* region. As a result, the p_1_–n–p_2_* structure was modified to become the p_1_*–n–p_2_* structure (4^th^ step: n–n–p_2_*- or i–n–p_2_*-like behaviour). After forming the BCCs in the both p-type layers, we found that the operation voltage was reduced below 0.5 V (4^th^ and 5^th^ steps) in the low-injection-current region (<±2.0 mA), indicating the conductive properties of p_1_*–n–p_2_*structure through the BCCs at the p_1_ and p_2_ regions (Fig. S3d and e). From these *I*–*V* results, we suggest that the p–n–p structure could form a n–n–p (or i–n–p) or p–n–n (p–n–i)-like structure by controlling the direction of the injection current after forming the BCCs in the p-layers.

**S4. AC-frequency dependent EL analysis of the BCC-embedded p_1_*–n–p_2_* LEDv**

**Figure S4** EL videos and images of the BCC-embedded p_1_*–n–p_2_* LEDv with different AC frequencies at a constant V_p_ of 7.0V. AC-frequencies applied to p_1_*–n–p_2_* LEDv are (**a**) 1Hz, (**b**) 10Hz, (**c**) 60Hz, and (**d**) 1000 Hz.

The light output power of p_1_*–n–p_2_* LEDv is well matched with the AC input-voltage shown in Fig. 5d. Fig. S4 exhibit the EL images and videos of p_1_*–n–p_2_* LEDv with different AC-frequencies. One can see that the light is alternately emitted from two p_1_* and p_2_* regions at 1.0 and 10 Hz AC frequencies shown in Fig. S4 a and b, respectively. At high AC frequencies above 60 Hz, the periodic luminescent interferences are observed in the videos due to the mismatch between the measured frequency of video and the emission frequency of LEDs. However, when viewed from the naked eye, we found that the two LEDs were continuously emitting light. In addition, we achieve that the p_1_*–n–p_2_* LEDv is well operating up to AC-frequency of 1.0 kHz. These results show that p_1_*–n–p_2_* LEDv can be applied to AC-illuminating light sources without AC-DC converters.
